# A scoring system based on artificial neural network for predicting 10-year survival in stage II A colon cancer patients after radical surgery

**DOI:** 10.18632/oncotarget.8217

**Published:** 2016-03-20

**Authors:** Jian-Hong Peng, Yu-Jing Fang, Cai-Xia Li, Qing-Jian Ou, Wu Jiang, Shi-Xun Lu, Zhen-Hai Lu, Pei-Xing Li, Jing-Ping Yun, Rong-Xin Zhang, Zhi-Zhong Pan, De-Sen Wan

**Affiliations:** ^1^ Department of Colorectal Surgery, Sun Yat-sen University Cancer Center, State Key Laboratory of Oncology in South China, Collaborative Innovation Center for Cancer Medicine Guangzhou, P.R. China; ^2^ Department of Colorectal Surgery, Department of Experimental Research, Sun Yat-sen University Cancer Center, State Key Laboratory of Oncology in South China, Collaborative Innovation Center for Cancer Medicine Guangzhou, P. R. China; ^3^ School of Mathematics and Computational Science, Sun Yat-sen University, Guangzhou, P.R. China; ^4^ Guangdong Provincial Key Laboratory of Computational Science, Sun Yat-sen University, Guangzhou, P.R. China; ^5^ Department of pathology, Sun Yat-sen University Cancer Center, State Key Laboratory of Oncology in South China, Collaborative Innovation Center for Cancer Medicine Guangzhou, P. R. China

**Keywords:** scoring system, artificial neural network, survival, stage IIA, colon cancer

## Abstract

Nearly 20% patients with stage II A colon cancer will develop recurrent disease post-operatively. The present study aims to develop a scoring system based on Artificial Neural Network (ANN) model for predicting 10-year survival outcome. The clinical and molecular data of 117 stage II A colon cancer patients from Sun Yat-sen University Cancer Center were used for training set and test set; poor pathological grading (score 49), reduced expression of TGFBR2 (score 33), over-expression of TGF-β (score 45), MAPK (score 32), pin1 (score 100), β-catenin in tumor tissue (score 50) and reduced expression of TGF-β in normal mucosa (score 22) were selected as the prognostic risk predictors. According to the developed scoring system, the patients were divided into 3 subgroups, which were supposed with higher, moderate and lower risk levels. As a result, for the 3 subgroups, the 10-year overall survival (OS) rates were 16.7%, 62.9% and 100% (*P* < 0.001); and the 10-year disease free survival (DFS) rates were 16.7%, 61.8% and 98.8% (*P* < 0.001) respectively. It showed that this scoring system for stage II A colon cancer could help to predict long-term survival and screen out high-risk individuals for more vigorous treatment.

## INTRODUCTION

Colorectal cancer (CRC) has been ranked as the fourth most common malignancies among women and the fifth among men in China [1]. For stage II colon cancer patients, the 5-year survival rate was approximately 80% after surgical resection [2-4]. According to the results of previous studies, 10% to 30% of these patients would eventually develop recurrent disease despite of receiving radical treatment [5, 6]. Several clinical indicators have been identified as risk factors including tumor stage T4, poor tumor grading, elevation of pre-operative carcino-embryonic antigen(CEA), presence of lymphovascular or perineural invasion [7-9]. Recently, certain molecular markers have been applied in predicting prognosis for stage II colon cancer. Lower-expression p53 was considered as a risk predictor of disease recurrence and bad survival outcome at 10 years [10]. Over-expression of survivin also significantly correlated with worse overall survival [11, 12]. However, any single clinical indicator or biomarker seems not of enough power to well predict the long-term survival outcome, and a predictive model by integrating both clinical indicators and molecular biomarkers is hopeful to meet the clinical needs.

Artificial neural network (ANN) is a mathematical and computational method that has been applied for diagnosis and prognosis prediction in several types of cancer in previous studies achieving higher sensitivity and specificity than the traditional procedures [13,14, 15]. The objective of this study is to construct a scoring system with ANN model to predict 10-year survival for stage II A colon cancer patients after radical surgery and then screen prognostic high-risk subgroup out of them.

## RESULTS

### Patient characteristics

Of the total 151 patients, complete results of Tissue Microarrays (TMA) analysis were obtained in 138 patients. With the median 12 years' follow-up, 21 patients (15.2%, 21/138) were lost to obtain a specific survival status. Thus, 117 cases (72 males and 45 females, with median age of 57 years) with definite survival outcome were included for analysis. Most of the tumors were moderately differentiated adenocarcinoma with median tumor size of 5 cm. Post-operative metastases occurred in 23 patients (19.6%, 23/117), mostly entailing liver metastases (12.8%, 15/117). Within 10-year follow-up, 16 patients (13.6%, 16/117) died of colon cancer progression. The 10-year overall survival (OS) and disease free survival (DFS) rate of total patients were both 86.8%. The 117 cases were randomly divided into a training set (*n* = 59) and an independent test set (*n* = 58). The clinical and pathological data are shown in Table 1.

**Table 1:** Clinical and pathological characteristics of total patients

	Training set	Test set	Total patients
Characteristics	(*n* = 59)	(*n* = 58)	(*n* = 117)
Gender			
Male	34(57.6%)	38(65.5%)	72(61.5%)
Female	25(42.4%)	20(34.5%)	45(38.5%)
Median age (year range)	56(24-84)	60(19-82)	57(19-84)
Mean Body Mass Index (BMI, kg/m^2^)	21.3±3.5	21.2±3.6	21.3±3.5
Median Tumor size(cm range)	5(2-18)	6(3-15)	5(2-18)
Tumor localization			
Right colon	25(42.4%)	20(34.5%)	45(38.5)
Left colon	34(57.6%)	38(65.5%)	72(61.5)
Adenocarcinoma type*			
Well-differentiated (Grade 1 and Grade 2)	48(81.4%)	48(82.8%)	96(82.0%)
Poorly-differentiated(Grade 3)	11(18.6%)	10(17.2%)	21(18.0%)
Median number of resected lymph nodes (range )	10(1-30)	6.5 (1-24)	8(1-30)
Postoperative metastatic site			
Liver	7(11.9%)	8(13.8%)	15(12.8%)
Lung	4(6.8%)	3(5.2%)	7(6.0%)
Bone	1(1.7%)	1(1.7%)	2(1.7%)
Brain	1(1.7%)	1(1.7%)	2(1.7%)
Abdominal and pelvic cavity	3(5.1%)	2(3.4%)	5(4.3%)

* Pathological grading was defined according to adenocarcinoma type

### ANN models and prognostic indicators

**Figure 1:**
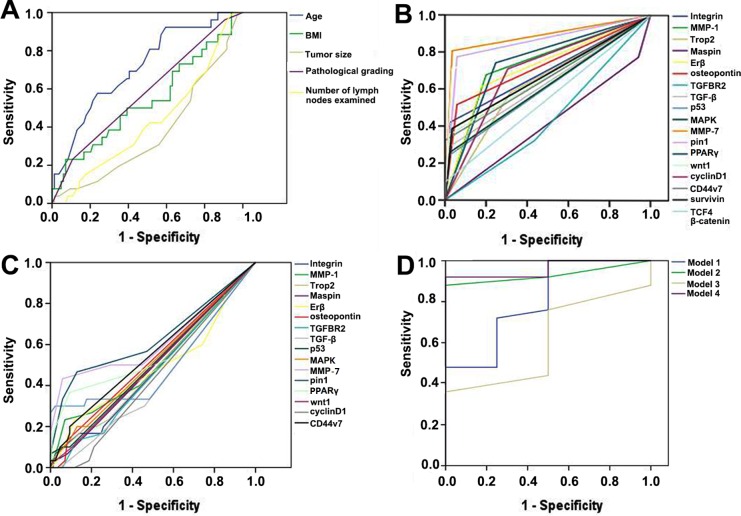
Receiver Operating Characteristic (ROC) curves represent **A.** clinical indicators of continuous and polytomous variables, **B.** tumor tissue biomarkers, **C.** normal mucosa biomarkers and **D.** Artificial Neural Networks (ANN) models separately.

Table 2 lists the cut-off points of binary classification for all clinical and molecular indicators and the results of univariate analyses on 10-year survival status. Figure 1 shows Receiver Operating Characteristic curve (ROC) for 5 clinical variables (Figure 1A), total molecular indicators in tumor tissue (Figure 1B) and those in normal mucosa (Figure 1C). We developed 4 ANN models. Model 1 started from 9 clinical indicators (Table 2). After analysis, tumor size, pathological grading, body mass index (BMI) and postoperative liver metastasis were selected as significant prognostic indicators. This model was moderately associated with 10-year survival outcome, with sensitivity 53.8%, specificity 97.8% and overall accuracy 87.9% (Figure 1D).

Model 2 started from 19 biomarkers in tumor tissue. As a result, TGFBR2, TGF-β, p53, MAPK, pin1 and β-catenin were identified as significant prognostic indicators. This model showed sensitivity 92.3%, specificity 91.1% and overall accuracy 91.4% (Figure 1D).

Since no variation was observed in survivin, TCF4, β-catenin in normal mucosa, Model 3 started from the rest 16 biomarkers, while TGFBR2, TGF-β, p53, MMP-7, pin1, PPARγ, wnt1 and cyclinD1 were recognized as the significant prognostic predictors. This model resulted in sensitivity 46.1%, specificity 82.2% and overall accuracy 74.1% (Figure 1D).

Model 4 integrated all the significant clinical indicators and biomarkers found by Model 1, Model 2 and Model 3. Pathological grading, TGFBR2, TGF-β, MAPK, pin1, β-catenin in tumor tissue and TGF-β in normal mucosa were ultimately identified as significant prognostic predictors. Model 4 was strongly correlated with the 10-year survival outcome, with sensitivity 92.3%, specificity 93.3% and overall accuracy 93.1% (Figure 1D).

**Table 2:** Univariate analysis and cut points of clinical indicators and molecular biomarkers

Variable	Hazard Ratio	95%CI	*p*
Clinical indicators			
Sex, female *vs* male	0.65	0.26 to 1.65	0.493
Age (years), ≥64 *vs* <64	3.81	1.54 to 9.43	0.006
BMI (kg/m2), ≥22.94 *vs* <22.94	2.16	0.82 to 5.66	0.181
Tumor localization, right *vs* left colon	0.45	0.15 to 1.62	0.241
Tumor size (cm), ≥6 *vs* <6	0.47	0.19 to 1.17	0.150
Pathological grading, ≥3 *vs* <3	2.73	0.87 to 8.57	0.150
Number of lymph nodes examined, ≥8 *vs* <8	0.38	0.15 to 0.94	0.057
Postoperative liver metastasis, yes *vs* no	44.50	9.00 to 220.06	<0.001
Postoperative lung metastasis, yes *vs* no	27.00	3.08 to 236.90	<0.001
Tumor tissue biomarkers			
Integrin ≥9 *vs* <9	44.50	9.00 to 220.06	<0.001
MMP 1 ≥9 *vs* <9	13.52	4.74 to 38.56	<0.001
Trop2 ≥5 *vs* <5	0.24	0.094 to 0.59	0.003
Maspin ≥9 *vs* <9	6.34	1.81 to 22.13	0.005
ERβ ≥4 *vs* <4	0.095	0.035 to 0.26	<0.001
Osteopontin ≥9 *vs* <9	19.32	6.20 to 60.17	<0.001
TGFBR2 ≥3 *vs* <3	0.59	0.23 to 1.50	0.378
TGF-β ≥9 *vs* <9	23.56	4.67 to 118.76	<0.001
p53 ≥10 *vs* <10	—	—	<0.001
MAPK ≥10 *vs* <10	19.78	3.87 to 100.96	<0.001
MMP7 ≥10 *vs* <10	131.87	29.32 to 593.06	<0.001
pin1 ≥9 *vs* <9	66.00	17.72 to 245.86	<0.001
PPARγ ≥8 *vs* <8	17.25	5.36 to 55.49	<0.001
wnt1 ≥8 *vs* <8	18.33	4.54 to 74.04	<0.001
CyclinD1 ≥5 *vs* <5	0.17	0.065 to 0.456	<0.001
CD44v7 ≥9 *vs* <9	—	—	<0.001
Survivin ≥9 *vs* <9	15.95	4.485 to 56.73	<0.001
TCF4 ≥12*vs* <12	—	—	0.010
β-catenin ≥9 *vs* <9	90.30	22.30 to 365.70	<0.001
Normal tissue biomarkers			
Integrin ≥2 *vs* <2	0.70	0.24 to 2.08	0.707
MMP 1 ≥2 *vs* <2	0.68	0.27 to 1.67	0.531
Trop2 ≥2 *vs* <2	0.62	0.21 to 1.81	0.528
Maspin ≥5 *vs* <5	—	—	0.502
ERβ ≥6 *vs* <6	2.43	0.84 to 6.99	0.170
Osteopontin ≥2 *vs* <2	1.35	0.33 to 5.52	0.966
TGFBR2 ≥5 *vs* <5	—	—	0.502
TGF-β ≥2 *vs* <2	0.57	0.22 to 1.44	0.327
p53 ≥2 *vs* <2	0.76	0.15 to 3.75	1
MAPK ≥5 *vs* <5	1.89	0.52 to 6.85	0.541
MMP-7 ≥4 *vs* <4	12.14	3.92 to 37.64	<0.001
pin1 ≥4 *vs* <4	5.14	1.95 to 13.56	0.001
PPARγ≥4 *vs* <4	4.29	1.51 to 12.15	0.01
wnt1 ≥2 *vs* <2	0.68	0.14 to 3.29	0.903
CyclinD1 ≥3 *vs* <3	—	—	0.031
CD44V7 ≥2 *vs* <2	1.47	0.42 to 5.15	0.79

**Abbreviations:** ERβ: Estrogen Receptor beta; MAPK: Mitogen-Activated Protein Kinase; Maspin: Matrix associated serine protease inhibitor; MMP-1: Matrix Metalloproteinase-1; MMP-7: Matrix Metalloproteinase-7; pin1: peptidyl prolylcis-trans isomerase 1; PPARγ: Peroxisome Proliferator-Activated Receptor-gamma; TGF4: Transforming Growth Factor 4; TGF-β: Transforming Growth Factor-beta; TGFBR2: Transforming Growth Factor-beta Receptor Type 2; Trop2: Tumor-associated calcium signal transducer-2; wnt1: wingless-type MMTV integration site family, member 1.

### Scoring system development

**Figure 2:**
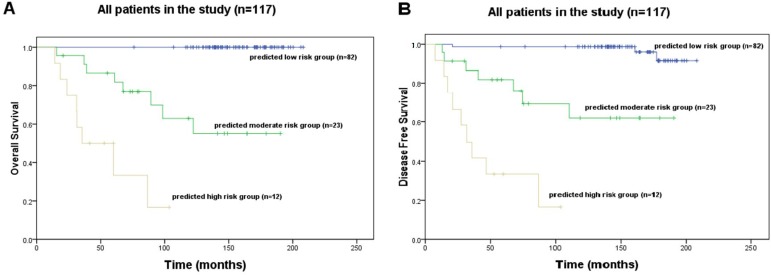
Kaplan-Meier survival curves show **A.** 10-year overall survival (OS) and **B.** 10-year disease free survival (DFS) of different subgroups from total patients.

The scoring system was developed based on 7 significant predictors in Model 4 (Table 3). In this system, 49 credits were assigned to poor pathological grading, 33 to reduced expression of TGFBR2, 45 to over-expression of TGF-β, 32 to over-expression of MAPK, 100 to over-expression of pin1, 50 to over-expression in tumor tissue and 22 to reduced expression of TGF-β in normal mucosa. For instance, a stage II A colon patient with a poor pathological grading, pin1 >9 and β-catenin >9 in tumor tissue would obtain a total score of 49+100+50 = 199. The relation between 10-year survival probability and the total score is shown in Table 4. Patients with total score ranging from 0 to 100 were classified into lower risk subgroup, 101 to 220 into moderate risk subgroup, and 221 to 331 into higher risk subgroup. The average 10-year OS rate in moderate risk subgroup was significantly larger than that in higher risk subgroup (62.9% *vs*. 16.7%; *P* < 0.001), but significantly smaller than that in lower risk subgroup (62.9% *vs*. 100%; *P* = 0.002) (Figure 2A); And similarly, the average 10-year DFS rate in moderate risk subgroup was significantly larger than that in higher risk subgroup (61.8% *vs*. 16.7%; *P* < 0.001), but significantly smaller than that in lower risk subgroup (61.8% *vs*. 98.8%; *P* = 0.003) (Figure 2B).

**Table 3:** Scores weighted by ANN analysis for each significant risk factor

		Standard score	
Risk factors	Threshold value	≥Threshold value	< Threshold value
Clinical factors Pathological grading Tumor tissue biomakers	3	49	0
TGFBR2	3	0	33
TGF-β	9	45	0
MAPK	10	32	0
pin1	9	100	0
β-catenin	9	50	0
Normal tissue biomakers			
TGF-β	2	0	22

**Abbreviations:** ANN: Artificial Neural Network; MAPK: Mitogen-Activated Protein Kinase; pin1: peptidyl prolylcis-trans isomerase 1; TGF-β: Transforming Growth Factor-beta; TGFBR2: Transforming Growth Factor-beta Receptor type 2.

**Table 4:** Correspondence between total scores and 10-year survival probability

Score	No. of patients	10-year survival probability	Risk classification
221-331	12	<20.0%	High risk
151-220	14	<50.0%	Moderate risk
101-150	9	<70%	Moderate risk
71-100	5	<80%	Low risk
31-70	47	<90%	Low risk
0-30	30	≤100%	Low risk

## DISCUSSION

Although 5-fluorouracil (5-FU)-based adjuvant chemotherapy is recommended for stage II B-C (T4N0) colon cancer patients, the necessity of post-operative chemotherapy in patients with stage II A colon cancer remains controversy [16-18]. Identification of the higher risk subgroup with poorer prognosis is a crucial strategy to optimize post-operative treatment for stage II A colon cancer patients. In current study, we developed a new approach to predict 10-year prognosis and screen out the higher risk individuals by integrating clinical factors and biomarkers into ANN model, which has not been exhibited in other studies yet.

TGF-β signaling pathway has been demonstrated to be one of common inactivated pathways in CRC, where TGFBR2 acts as a metastatic suppressor [19, 20]. Additionally, previous studies have showed that TGFBR2 was a prognostic marker and its down regulation exhibited poor overall survival in oral cancer and breast cancer [21, 22]. In our data, TGFBR2 was also served as a protective prognostic factor for stage II A colon cancer. However, we found an opposite predicting result of TGF-β expression between tumor tissue and normal mucosa of stage II A colon cancer. Our study shows that over-expression of TGF-β in tumor tissue is a high risk factor for long-term survival, whereas over-expression of TGF-β in normal mucosa is a protective prognostic factor. It is noted that TGF-β has bidirectional functions in the progression of cancer: it acts as a tumor suppressor during the early stages by inhibiting cell proliferation while it functions as a pro-oncogenic factor during tumor progression through stimulating induction of epithelial-mesenchymal transition (EMT) [23, 24].

MAPK signaling pathway is correlated with proliferation, survival and apoptosis of tumor cells, and therefore activates the carcinogenesis [25]. Zolota et al. found high expression of MAPK signaling pathway components indicated poor overall survival of patients with gliomas, suggesting that MAPK attributed to promoting biologic behavior of gliomas [26]. Similarly, Yu et al. have reported that high MAPK expression in colorectal cancer specimen predicted poorer prognosis [27]. Nevertheless, Yu et al. did not assess MAPK expression in normal colon tissue of the patients. By evaluating the staining image of immunohistochemistry (IHC) in both tumor and normal tissue, our model indicated that MAPK expression in tumor tissue instead of normal tissue was negatively correlated to better long-term outcome in stage II A colon cancer.

Pin1 has been reported that it contributed to tumor development and promoted tumor aggressiveness [28]. Its over-expression is related with poorer survival outcome in gastric cancer and non-small cell lung cancer [29, 30]. Additionally, Kuramochi et al. found that high expression of pin1 was correlated with poor histological type (*P* = 0.0240), more aggressive invasion (*P* = 0.0051), and worse staging (*P* = 0.0027) of colorectal tumors [31]. In present study, we observed a strong correlation between pin 1 expression and survival outcome, which inferenced pin1 expression as an excellent prognostic predictor for stage II A colon cancer. On the other hand, it has been widely reported that pin 1 could upregulate β-catenin expression in tumor progression [31-33]. As a vital member of Wnt/β-catenin pathway, β-catenin activates tumorigenic behaviors, such as migration, stemness, anchorage-independent growth and chemo-sensitivity [34]. However, the prognostic significance of β-catenin expression in CRC remains unclear. Previous studies as well as our study showed the worse survival outcome for patients with high expression of nuclear β-catenin [35, 36], whereas some showed that the high nuclear expression was associated with a better prognosis [37, 38], and other studies even failed to find any predictive value of nuclear β-catenin in CRC [39, 40]. Further studies are needed to verify the predictive value of β-catenin in CRC.

The scoring system we have constructed can convert the result of ANN models into a visualized scale, thus making it easier to calculate the probability for 10-year survival. Further more, the subgroups defined by this scoring system revealed that the patients in subgroup with higher score had a poorest long-term prognosis, thus proving its strong discriminate power. The system will be helpful for making clinical decision by guiding post-operative treatment for the higher risk subgroup, such as adjuvant chemotherapy and more normative follow-up.

Several potential limitations of this scoring system should be acknowledged. First of all, only the final survival status after 10 years post-operatively was investigated, while the time dimension was not taken into account. Second, the complete data of post-operative treatment for patients failed to be obtained, which impeded us to predict the long-term survival outcome by post-operative treatment factors. Post-operative treatment regimens and duration were distinguished in several patients, which might impair the analysis accuracy without concerning of the situation. Third, since this scoring system was generated based on a limited number of patients from a single institution, the validity of this scoring system should be improved by large-volume and prospective studies.

In conclusion, this scoring system based on ANN model have identified reduced expression of TGFBR2, over-expression of TGF-β, MAPK, pin1, β-catenin in tumor tissue and reduced expression of TGF-β in normal mucosa as significant risk predictors for 10-year survival outcome in stage II A colon cancer patients after radical surgery. We considered this scoring system can better predict long-term survival outcome and then screen out high-risk individuals with stage II A colon cancer for further aggressive post-operative treatment.

## MATERIALS AND METHODS

### Patient selection

The study included 151 patients with stage II A colon cancer who underwent lesions radical resection from August 1996 to May 2003 at Department of Colorectal Surgery in Sun Yat-sen University Cancer center. The detailed clinical information of the eligible patients was collected through the electronic medical records system. The inclusion criteria were: (1) The histological diagnosis should be proven as colon adenocarcinomas; (2) The disease stage should be confirmed as T3N0M0 according to 2010 American Joint Committee on Cancer/International Union Against Cancer staging system (AJCC/UICC); (3) No adjuvant therapy was received by any patient; (4) No evidence showed any history of other active malignancy (except for basal cell carcinoma of the skin) and existence of multiple primary colorectal cancer. All patients were recommended to follow up every 6 months for 10 years post-operatively.

The study was approved by the Medical Ethics Committee of the Cancer Center, Sun Yat-sen University (NO. GZR2011-10).

### Tissue microarrays (TMA) construction and immunohistochemistry (IHC) scoring

We used a tissue array instrument (personal tissue arrayer, Beeche, USA) to convert the paraffin-embedded specimens of individual tumor and normal mucosa into TMA construction. TMA and IHC were developed based on the methods described in our previous study [41]. Anti-human antibody of 19 prognostic markers involved in colon cancer progression were chosen for incubating the TMA slice, including Integrin (1: 100, monoclonal mouse, Abcam), Matrix Metalloproteinases-1 (MMP-1, 1: 250, polyclonal rabbit, Abcam), Matrix Metalloproteinases-7 (MMP-7, 1: 250, monoclonal rabbit, Cell Signaling Technology), Tumor-associated calcium signal transducer-2 (Trop2, 1:200, polyclonal rabbit, Thermo Scientific), Matrix associated serine protease inhibitor (Maspin, 1:1000, polyclonal rabbit, Abcam), Estrogen Receptor beta (ERβ,1:500, monoclonal rabbit, Abcam), osteopontin (1:200, polyclonal rabbit, Sigma-Aldrich), Transforming Growth Factor-beta (TGF-β, 1: 100, polyclonal rabbit, Abcam), Transforming Growth Factor-beta Receptor type 2 (TGFBR2, 1: 100, monoclonal mouse, Abcam), p53(1:150, monoclonal mouse, Cell Signaling Technology), Mitogen-Activated Protein Kinase (MAPK, 1:50, monoclonal rabbit, Abcam), Peptidyl prolylcis-trans isomerase-1 (pin1, 1:400, polyclonal rabbit, Abcam), Peroxisome proliferator-activated receptor gamma (PPARγ, 1:200, polyclonal rabbit, Abcam), wingless-type MMTV integration site family, member 1(wnt1, 1:200, polyclonal rabbit, Thermo Scientific), cyclinD1 (1: 50, monoclonal rabbit, Cell Signaling Technology), CD44v7 (1: 400, monoclonal mouse, Abcam), Survivin (1: 400, monoclonal rabbit, Cell Signaling Technology), Transcription Factor 4 (TCF4, 1:50, polyclonal rabbit, Abcam) and β-catenin (1: 100, monoclonal rabbit, Cell Signaling Technology). The slides were counter stained with Mayer's hematoxylin and observed in Olmpus Scaper.

Each slide was evaluated by applying IHC scoring system. The positively-stained was measured as follows: “0”(less than 5% positively-stained cells), “1” (6-24% of positively-stained cells), “2” (25-49% of positively stained cells), “3” (50-74% of positively-stained cells) and “4” (75%-100% of positively-stained cells). The intensity was measured according to the standard: “0” (negative staining); “1” (weak staining); “2” (moderate staining) and “3” (strong staining). The final value was obtained by summarizing the above two values. Two trained pathologists independently evaluated all cases blindly. If the conclusion of two pathologists was controversial, then the process was repeated to find a common result.

### Statistical analysis

In this study, each continuous or ordinal variable was dichotomized by a cut-off point chosen *via* ROC analysis and then went through a univariate analysis for 10-year survival outcome. The ANN applied in this study was a Multi-layer Perceptron (MLP) neural network including three layers: an input layer, a hidden layer and an output layer. The first layer has input neurons which sends data *via* synapses to the hidden layer of neurons, and then *via* more synapses to the third layer of output neurons. ANN was constructed to predict 10-year survival status using R packages, including “caret” and “nnet” for ANN model selection with recursive backwards feature selection *via* bootstrap resampling technique, “pROC” for ROC analysis and “rms” for scoring system development. Basic ANN model of clinical indicators and biomarkers were generated respectively, which was subsequently integrated to establish an ultimate model. The data of each ANN model was randomly and equally distributed into training set and test set by sample function of R packages. All ANN models were modeled with training set and evaluated their accuracy with test set. Scoring system was developed by converting the ultimate ANN model into principal component regression model with predicted values from the ANN model as responses. The score for each risk factor was assigned according to the regression coefficient. The total score for each individual was calculated by adding the allocated scores corresponding to a certain 10-year survival probability. According to the distinguishing survival probability, the patients were divided into distinct risk groups and the difference of 10-year OS and DFS were compared among groups by applying Log-rank test respectively.

## References

[1] Chen W, Zheng R, Zeng H, Zhang S, He J (2015). Annual report on status of cancer in China, 2011. Chinese journal of cancer research.

[2] Group QC (2007). Adjuvant chemotherapy *versus* observation in patients with colorectal cancer: a randomised study. The Lancet.

[3] Santos C, Lopez-Doriga A, Navarro M, Mateo J, Biondo S, Villacampa MM, Soler G, Sanjuan X, Paules MJ, Laquente B, Guino E, Kreisler E, Frago R, Germa JR, Moreno V, Salazar R (2013). Clinical risk factors of Stage II colon cancer: results of a prospective study. Colorectal Disease.

[4] de Gramont A, Hubbard J, Shi Q, O'Connell MJ, Buyse M, Benedetti J, Bot B, O'Callaghan C, Yothers G, Goldberg RM, Blanke CD, Benson A, Deng Q, Alberts SR, Andre T, Wolmark N (2010). Association Between Disease-Free Survival and Overall Survival When Survival Is Prolonged After Recurrence in Patients Receiving Cytotoxic Adjuvant Therapy for Colon Cancer: Simulations Based on the 20,800 Patient ACCENT Data Set. Journal of Clinical Oncology.

[5] Hutchins G, Southward K, Handley K, Magill L, Beaumont C, Stahlschmidt J, Richman S, Chambers P, Seymour M, Kerr D, Gray R, Quirke P (2011). Value of Mismatch Repair, KRAS, and BRAF Mutations in Predicting Recurrence and Benefits From Chemotherapy in Colorectal Cancer. Journal of Clinical Oncology.

[6] Tsikitis VL, Larson DW, Huebner M, Lohse CM, Thompson PA (2014). Predictors of recurrence free survival for patients with stage II and III colon cancer. BMC Cancer.

[7] Quah HM, Chou JF, Gonen M, Shia J, Schrag D, Landmann RG, Guillem JG, Paty PB, Temple LK, Wong WD, Weiser MR (2008). Identification of patients with high-risk stage II colon cancer for adjuvant therapy. Diseases of the colon and rectum.

[8] Yamaguchi K, Ogata Y, Akagi Y, Shirouzu K (2013). Identification of high-risk factors as indicators for adjuvant therapy in stage II colon cancer patients treated at a single institution. Oncology letters.

[9] Gertler R, Rosenberg R, Schuster T, Friess H (2009). Defining a high-risk subgroup with colon cancer stages I and II for possible adjuvant therapy. Eur J Cancer.

[10] Resnick MB, Routhier J, Konkin T, Sabo E, Pricolo VE (2004). Epidermal Growth Factor Receptor, c-MET, β-Catenin, and p53 Expression as Prognostic Indicators in Stage II Colon Cancer: A Tissue Microarray Study. Clinical Cancer Research.

[11] Huang YJ, Qi WX, He AN, Sun YJ, Shen Z, Yao Y (2013). The prognostic value of survivin expression in patients with colorectal carcinoma: a meta-analysis. Japanese journal of clinical oncology.

[12] Krieg A, Werner TA, Verde PE, Stoecklein NH, Knoefel WT (2013). Prognostic and clinical significance of survivin in colorectal cancer: a meta-analysis. PloS one.

[13] Spelt L, Nilsson J, Andersson R, Andersson B (2013). Artificial neural networks—a method for prediction of survival following liver resection for colorectal cancer metastases. European journal of surgical oncology.

[14] Wu Y, Wu Y, Wang J, Yan Z, Qu L, Xiang B, Zhang Y (2011). An optimal tumor marker group-coupled artificial neural network for diagnosis of lung cancer. Expert Systems with Applications.

[15] Shi HY, Lee KT, Wang JJ, Sun DP, Lee HH, Chiu CC (2012). Artificial neural network model for predicting 5-year mortality after surgery for hepatocellular carcinoma: a nationwide study. Journal of gastrointestinal surgery.

[16] Benson AB, Schrag D, Somerfield MR, Cohen AM, Figueredo AT, Flynn PJ, Krzyzanowska MK, Maroun J, McAllister P, Van Cutsem E, Brouwers M, Charette M, Haller DG (2004). American Society of Clinical Oncology recommendations on adjuvant chemotherapy for stage II colon cancer. Journal of clinical oncology.

[17] Gray R, Barnwell J, McConkey C, Hills RK, Williams NS, Kerr DJ, Quasar Collaborative G (2007). Adjuvant chemotherapy *versus* observation in patients with colorectal cancer: a randomised study. Lancet.

[18] Zhao S, Jiang T, Tang H, Cui F, Liu C, Guo F, Lu H, Xue Y, Jiang W, Peng Z, Yan D (2015). Ubiquitin D is an independent prognostic marker for survival in stage IIB-IIC colon cancer patients treated with 5-fluoruracil-based adjuvant chemotherapy. Journal of gastroenterology and hepatology.

[19] Yu M, Trobridge P, Wang Y, Kanngurn S, Morris SM, Knoblaugh S, Grady WM (2014). Inactivation of TGF-beta signaling and loss of PTEN cooperate to induce colon cancer *in vivo*. Oncogene.

[20] Zhang W, Zhang T, Jin R, Zhao H, Hu J, Feng B, Zang L, Zheng M, Wang M (2014). MicroRNA-301a promotes migration and invasion by targeting TGFBR2 in human colorectal cancer. Journal of experimental & clinical cancer research : CR.

[21] Sivadas VP, Saakshi G, Iype EM, Balan A, Kannan S (2015). Prognostic implication of the loss of TGFBR2 expression in oral carcinoma. Neoplasma.

[22] Busch S, Acar A, Magnusson Y, Gregersson P, Ryden L, Landberg G (2015). TGF-beta receptor type-2 expression in cancer-associated fibroblasts regulates breast cancer cell growth and survival and is a prognostic marker in pre-menopausal breast cancer. Oncogene.

[23] Slabakova E, Pernicova Z, Slavickova E, Starsichova A, Kozubik A, Soucek K (2011). TGF-beta 1-Induced EMT of Non-Transformed Prostate Hyperplasia Cells Is Characterized by Early Induction of SNAI2/Slug. Prostate.

[24] Pignatelli J, Tumbarello DA, Schmidt RP, Turner CE (2012). Hic-5 promotes invadopodia formation and invasion during TGF-beta-induced epithelial-mesenchymal transition. J Cell Biol.

[25] Platanias LC (2003). Map kinase signaling pathways and hematologic malignancies. Blood.

[26] Zolota V, Sirinian C, Kefalopoulou Z, Panagiotopoulos V, Spinos P, Argyriou AA, Kalofonos HP (2013). Mitogen-activated protein kinases in gliomas and correlation with patients' prognosis. Acta neurologica Scandinavica.

[27] Yu SJ, Yu JK, Ge WT, Hu HG, Yuan Y, Zheng S (2011). SPARCL1, Shp2, MSH2, E-cadherin, p53, ADCY-2 and MAPK are prognosis-related in colorectal cancer. World journal of gastroenterology : WJG.

[28] Girardini Javier E, Napoli M, Piazza S, Rustighi A, Marotta C, Radaelli E, Capaci V, Jordan L, Quinlan P, Thompson A, Mano M, Rosato A, Crook T, Scanziani E, Means Anthony R, Lozano G (2011). A Pin1/Mutant p53 Axis Promotes Aggressiveness in Breast Cancer. Cancer Cell.

[29] Shi M, Chen L, Ji J, Cai Q, Yu Y, Liu B, Zhu Z, Zhang J (2015). Pin1 is Overexpressed and Correlates with Poor Prognosis in Gastric Cancer. Cell Biochem Biophys.

[30] Tan X, Zhou F, Wan J, Hang J, Chen Z, Li B, Zhang C, Shao K, Jiang P, Shi S, Feng X, Lv N, Wang Z, Ling Y, Zhao X, Ding D (2010). Pin1 expression contributes to lung cancer prognosis and carcinogenesis. Cancer Biology & Therapy.

[31] Kuramochi J, Arai T, Ikeda S, Kumagai J, Uetake H, Sugihara K (2006). High Pin1 expression is associated with tumor progression in colorectal cancer. J Surg Oncol.

[32] Lin FC, Lee YC, Goan YG, Tsai CH, Yao YC, Cheng HC, Lai WW, Wang YC, Sheu BS, Lu PJ (2014). Pin1 positively affects tumorigenesis of esophageal squamous cell carcinoma and correlates with poor survival of patients. Journal of biomedical science.

[33] Nakamura K, Kosugi I, Lee DY, Hafner A, Sinclair DA, Ryo A, Lu KP (2012). Prolyl Isomerase Pin1 Regulates Neuronal Differentiation *via* β-Catenin. Molecular and cellular biology.

[34] Xu J, Prosperi JR, Choudhury N, Olopade OI, Goss KH (2015). β-Catenin Is Required for the Tumorigenic Behavior of Triple-Negative Breast Cancer Cells. PloS one.

[35] Youssef NS, Osman WM (2015). Relationship between osteopontin and β-catenin immunohistochemical expression and prognostic parameters of colorectal carcinoma. International Journal of Clinical and Experimental Pathology.

[36] Stanczak A, Stec R, Bodnar L, Olszewski W, Cichowicz M, Kozlowski W, Szczylik C, Pietrucha T, Wieczorek M, Lamparska-Przybysz M (2011). Prognostic Significance of Wnt-1, β-catenin and E-cadherin Expression in Advanced Colorectal Carcinoma. Pathol Oncol Res.

[37] Elzagheid A, Buhmeida A, Korkeila E, Collan Y, Syrjanen K, Pyrhonen S (2008). Nuclear beta-catenin expression as a prognostic factor in advanced colorectal carcinoma. World journal of gastroenterology : WJG.

[38] Wangefjord S, Brändstedt J, Lindquist KE, Nodin B, Jirström K, Eberhard J (2013). Associations of beta-catenin alterations and MSI screening status with expression of key cell cycle regulating proteins and survival from colorectal cancer. Diagnostic Pathology.

[39] Wanitsuwan W, Kanngurn S, Boonpipattanapong T, Sangthong R, Sangkhathat S (2008). Overall expression of beta-catenin outperforms its nuclear accumulation in predicting outcomes of colorectal cancers. World journal of gastroenterology : WJG.

[40] Bruun J, Kolberg M, Nesland JM, Nesbakken A, Svindland A, Lothe RA (2014). Prognostic significance of β-catenin, E-cadherin and SOX9 in colorectal cancer: results from a large population-representative series. Frontiers in Oncology.

[41] Hu W, Lu SX, Li M, Zhang C, Liu LL, Fu J, Jin JT, Luo RZ, Zhang CZ, Yun JP (2015). Pyruvate kinase M2 prevents apoptosis *via* modulating Bim stability and associates with poor outcome in hepatocellular carcinoma. Oncotarget.

